# The 26S Proteasome and Initiation of Gene Transcription

**DOI:** 10.3390/biom4030827

**Published:** 2014-09-10

**Authors:** Geetha Durairaj, Peter Kaiser

**Affiliations:** Department of Biological Chemistry, University of California, Irvine, CA 92697, USA; E-Mail: gduraira@uci.edu

**Keywords:** ubiquitin, E3 ligase, proteasome, transcription, APIS

## Abstract

Transcription activation is the foremost step of gene expression and is modulated by various factors that act in synergy. Misregulation of this process and its associated factors has severe effects and hence requires strong regulatory control. In recent years, growing evidence has highlighted the 26S proteasome as an important contributor to the regulation of transcription initiation. Well known for its role in protein destruction, its contribution to protein synthesis was initially viewed with skepticism. However, studies over the past several years have established the proteasome as an important component of transcription initiation through proteolytic and non-proteolytic activities. In this review, we discuss findings made so far in understanding the connections between transcription initiation and the 26S proteasome complex.

## 1. Introduction

Evolution from prokaryotes to eukaryotes has led to well-organized complexity in many aspects of biology, and gene expression is no exception. In contrast to the situation in prokaryotes, gene expression in eukaryotes is a compartmentalized process consisting of various steps including transcription and processing of transcripts inside the nucleus, and subsequent translation into proteins in the cytoplasm. Transcription is divided into different phases: transcription initiation, elongation, and termination [1,2,3]. All these steps are highly interconnected via an extensive network of physical and functional interactions. To coordinate these steps, cells employ various regulatory mechanisms, especially at the level of transcription initiation. The proteasome plays pivotal roles in all steps of transcription and is important in controlling both the magnitude and temporal aspects of gene expression. This review will only address the role of the proteasome in initiation of transcription. The reader is referred to excellent reviews describing functions of the proteasome in other aspects of gene expression [4,5,6,7,8].

Transcription initiation typically involves the binding of an activator protein to a specific DNA sequence via an intrinsic or a co-factor-provided DNA binding domain. The promoter-bound activation domain facilitates recruitment of general transcription factors (GTFs) TFIIA, TFIIB, TFIID, TFIIE, TFIIF, TFIIH, RNA polymerase II holoenzyme, and chromatin-remodeling complexes to form the *Pre-Initiation Complex* (PIC) [9,10,11,12]. Formation of a PIC marks initiation of transcription and is followed by transcription elongation and termination. With the assembly, and often dynamic interactions, of dozens of factors on a given promoter, mechanisms of temporal and spatial control of these assemblies remain largely unknown. In recent years, the 26S proteasome has emerged as an important regulator of transcription initiation [13,14,15,16,17]. Some proteasome roles in transcription are obvious, and one can easily comprehend how proteasome-mediated degradation of transactivator or repressor proteins influences gene transcription. However, a number of studies have identified functions of the proteasome in activation of transcription through activator degradation, which may seem counterintuitive and was initially viewed with skepticism. The multifaceted ways cells engage proteasomes to control gene expression emphasizes once again the central place of this remarkable protein assembly in cellular processes.

The 26S proteasome is a non-lysosomal proteolytic machine that consists of two sub-complexes: the 20S proteolytic core particle (CP) and the 19S regulatory particle (RP) [18,19]. The proteolytic activity resides within the 20S CP, while the 19S RP confers specificity to ubiquitin bound substrates and ATP-dependent protein unfolding. The 20S CP has a cylinder-like structure that is composed of four rings (2α and 2β) in the order α-β-β-α. Three of the seven β subunits possess chymotrypsin-like, trypsin-like, and caspase-like activities [20,21]. The 19S RP is composed of two subcomplexes: the base and lid. The lid consists of nine non-ATPases while the base contains a hetero-hexameric ring of AAA (ATPases associated with a variety of cellular activities) family ATPases and four non-ATPases [21,22]. Substrates are typically recognized by 19S lid components through substrate-attached polyubiquitin chains, followed by removal of the ubiquitin chain, unfolding catalyzed by ATPases, and translocation into the 20S core cavity for degradation. Although beyond the scope of this review, an alternative form of the 20S proteasome known as the immunoproteasome is formed by the replacement of catalytic β1, β2 and β5 subunits by interferon-γ-inducible subunits β1i (LMP2), β2i (MECL1), and β5i (LMP7), respectively [23,24]. The resulting enhanced chymotrypsin-like activity facilitates the production of peptides for presentation by MHC class I. Initially discovered because of its role in inflammation-triggered peptide processing, research over the past several years has shown the immunoproteasome’s involvement in mediating a variety of cellular processes including transcription [24,25]. However, the role of the immunoproteasome in transcription is just beginning to emerge and mechanistic aspects await discovery.

The 26S proteasome degrades proteins that are marked with ubiquitin chains, which are attached by the E1-E2-E3 enzyme cascade [26]. Typically at least four or more ubiquitin moieties must be attached to substrate proteins in order to target them for proteasomal degradation. Monoubiquitylation can, in some cases, be sufficient for proteasome-mediated degradation [27,28,29], but more often presents a non-proteolytic signal involved in DNA repair, endocytosis, endosomal sorting, histone regulation, and mRNA export [22,26,30]. The fate of a polyubiquitylated protein depends on the topology of the attached ubiquitin chain. While ubiquitin chains formed by linkages involving lysine in position 48 of ubiquitin (K48-chains) represent the canonical proteasome targeting signal, K63-linked chains have been shown to regulate a variety of non-proteolytic cellular functions [31,32]. However, exceptions to these general concepts are well established [33,34,35,36,37,38], suggesting that ubiquitin chains can trigger multiple signals in coordinated temporal succession. However, analyses are too often focused on the most visible output, proteasome-mediated degradation. Much less is known about non-canonical ubiquitin chains linked through one of the five other lysine residues of ubiquitin, or the amino terminal methionine [26,35]. Most non-canonical chain topologies are targeting signals for the proteasome, but may modulate efficiency of proteasome binding, chain processing, or target protein degradation and may thus play unrecognized roles in fine-tuning of transcription. We are only beginning to understand the complexity of the ubiquitin code. Future research will define how ubiquitin chain types modulate the role of the proteasome at transcription sites.

## 2. The 26S Proteasome in Transcription Activation

First indications of a role of proteasomes in transcriptional activation emerged when Finley and colleagues [39] identified the presumed RNA polymerase II component Sug1 (subsequently named Rpt6 [40]) as a core subunit of the budding yeast 19S proteasome. Mutations in *sug1* were originally identified as suppressors of transcription defects caused by *gal4* transactivation domain (TAD) mutations [41]. Subsequently, a second yeast 19S subunit, Sug2/Rpt4, with similar genetic interactions with the yeast activator Gal4 was characterized [42]. These initial findings suggested that proteolytic events are critical for transcription activation. However, mutations in 20S subunits, which block the proteolytic functions of the proteasome, did not suppress the defects of *gal4* TAD mutants [43]. Other activities of the proteasome or its sub-complexes might thus be involved in transcription regulation. However, the identification of a striking and widespread overlap between transcription activation domains (TADs) and degradation signals (degrons) in most unstable transcription factors not only substantiated the role of proteasome in transcription activation, but also suggested direct participation of protein degradation [44]. These findings hinted at complex and diverse roles of proteasomes in transcription regulation that engage the different activities housed in the 26S proteasome complex.

With the evidence from studies that followed [13,16,17,45,46,47,48,49], it is now clear that the 26S proteasome is involved in controlling interactions of transcription initiation factors, their abundance, and their localization. Genome-wide location analyses showed that proteasomes associate with majority of yeast genes [50,51], suggesting a fundamental connection of transcription and proteasomes. Genome-wide expression analysis in yeast in response to either genetic or chemical inhibition of proteasome function demonstrated significant changes in gene expression patterns [7,50,52,53]. Genes involved in mitochondrial function, stress response, and protein degradation were upregulated, while ribosomal protein genes, mating, and amino acid metabolism genes were down-regulated. Such studies were also extended to human cells and inhibition of proteasomes with bortezomib had a drastic effect on regulation of the majority of genes [54]. While it is tempting to assume a role of the 26S proteasome in transcription initiation from these global studies, a direct connection cannot be concluded. The proteasome controls abundance of many proteins and its inhibition will affect signaling networks leading to indirect effects on transcription [55]. An indirect consequence of proteasome inhibition is the rapid mobilization of ubiquitin from ubiquitylated histones to compensate for depletion of ubiquitin monomers, which are trapped in stabilized polyubiquitin conjugates [56,57]. The resulting widespread loss of ubiquitylated histones can have a significant but indirect effect on transcription. Furthermore, specific effects on initiation cannot be deduced from mRNA abundance changes. More directed approaches will need to be used to examine the role of the proteasome in initiation.

### 2.1. Proteolytic Function of the 26S Proteasome in Transcription Activation

**Figure 1:**
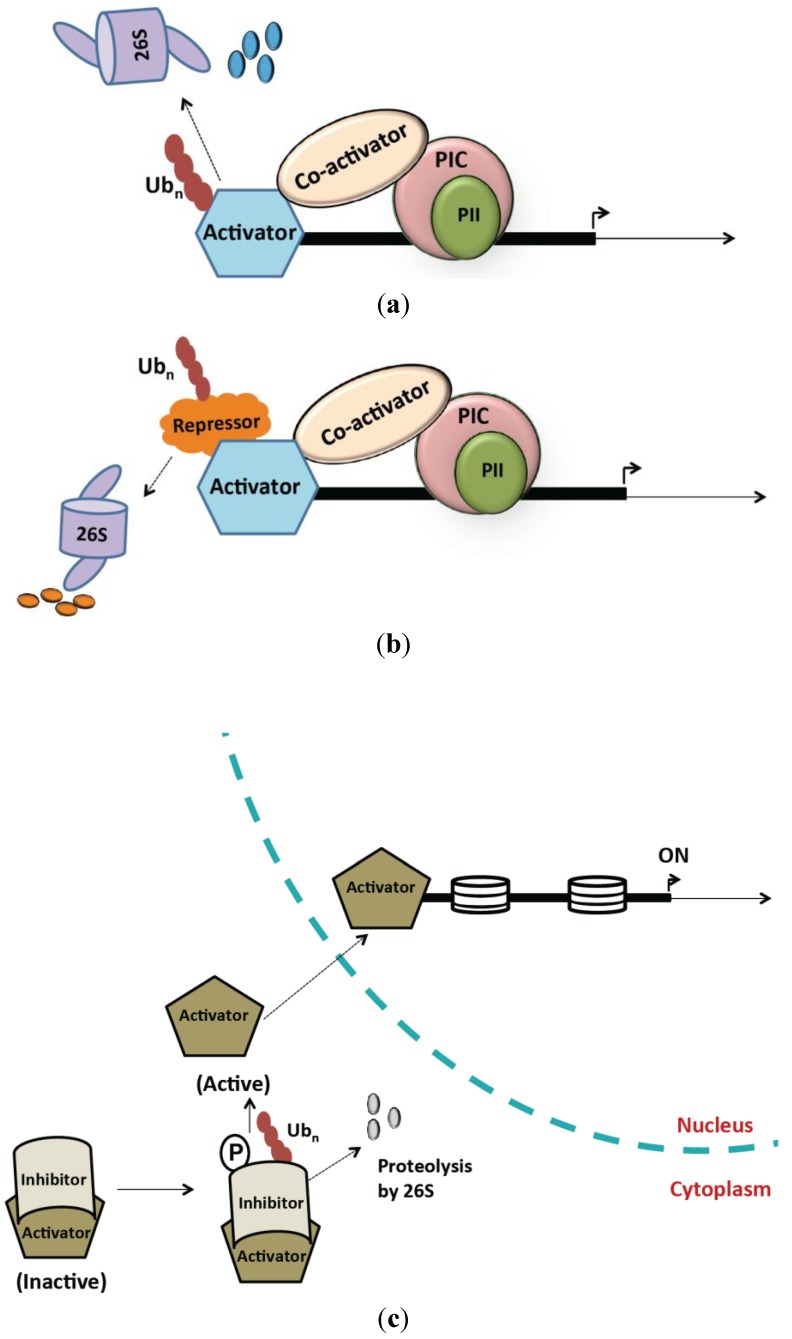
Proteolysis-dependent roles of the 26S proteasome in transcription activation. (**a**) Activator abundance is regulated by the 26S proteasome. Activator degradation can be transcription dependent where the initiation of transcription marks the activator for destruction. Alternatively, transcription independent degradation prevents initiation until activator stabilization (see main text for details); (**b**) The 26S proteasome is involved in degradation of transcriptional repressors thereby activating transcription; (**c**) Inhibitor-bound activators can be sequestered in a dormant state in the cytoplasm and the 26S proteasome regulates the destruction of the inhibitor thereby promoting the relocalization of the activator to the nucleus.

Activation of transcription starts with binding of an activator protein to promoters to stimulate formation of various protein complexes. Mostly, activators are held in inactive forms and switch to active states in response to cellular cues. This switch can be mediated by disruption of activator-inhibitor interactions, control of activator abundance, or through subcellular localization of activators. The 26S proteasome complex controls initiation through all of these mechanisms as discussed below and depicted in Figure 1.

#### 2.1.1. Activator Ubiquitylation and Turnover

Binding of activators to DNA sequences is essential for transcriptional activity, but timely repression of activators is equally important. Cells employ ubiquitin-dependent proteolysis of activators to either limit activation or turn off transcription. Surprisingly, proteolysis of activators has also been shown to stimulate their function. These findings have led to the suggested classification of transcriptional activators into three majors classes A, B and C, depending on their functional interaction with the ubiquitin proteasome system [43].

**Figure 2:**
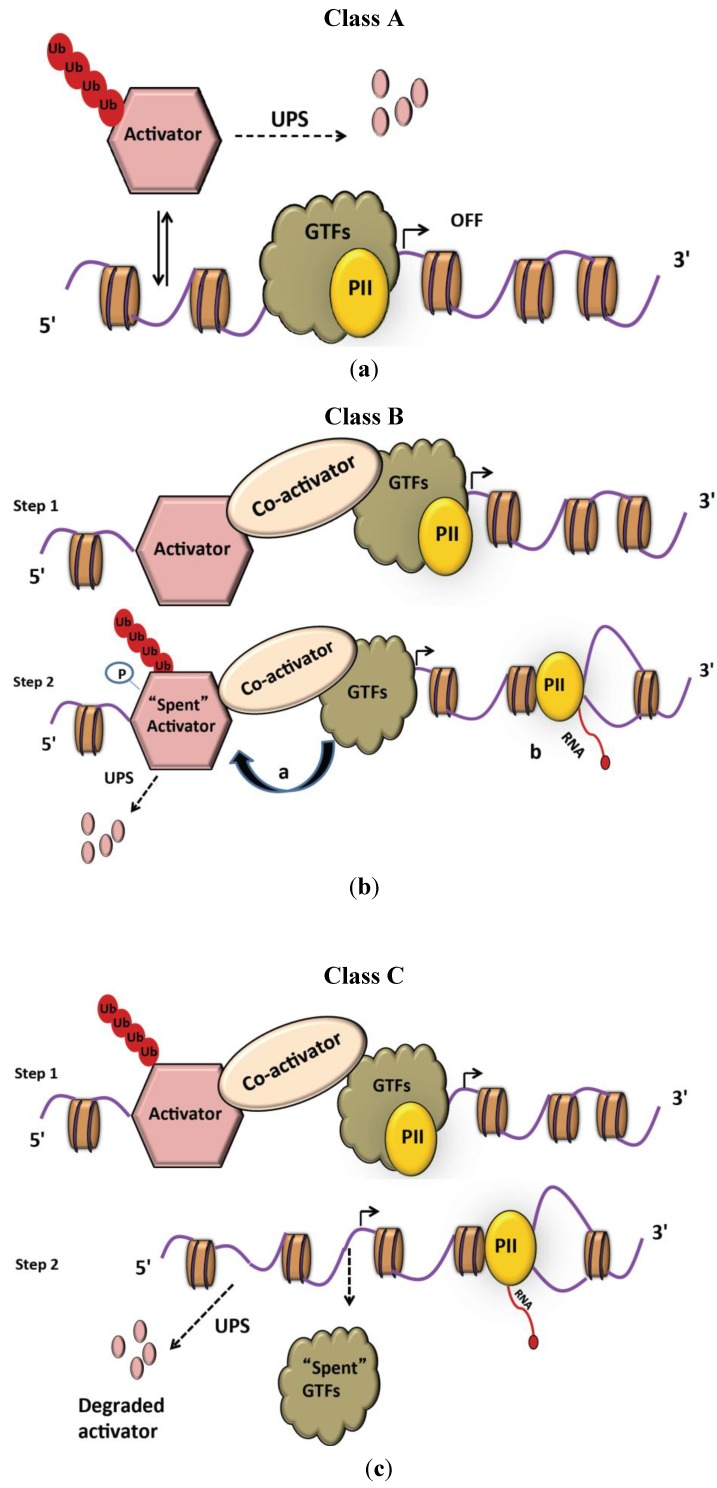
Classification of transcriptional activators based on their proteasomal degradation patterns. (**a**) Class A activators are degraded when they are not bound to DNA. The stability of these activators dictates the balance between activation and repression of transcription; (**b**) Class B activators bind to DNA and promote the association of GTFs with DNA. Such activation marks the activator as “spent”, often by phosphorylation. GTFs in turn recruit E3 ligase(s) that ubiquitylate the “spent” activator leading to its degradation and thereby facilitating ongoing transcription; (**c**) Class C activators are a specialized form of class B, whose degradation serves as a signal for initiation. Once the GTFs are assembled, the activator is degraded, which in turn destabilizes the “spent” GTFs to allow the escape of phosphorylated RNAPII to facilitate transcription.

Turnover of class A activators does not depend on transcription and occurs when the factors are not bound to DNA (Figure 2a). One of the well-studied activators of this class is p53. The tumor suppressor protein p53 is a transcription factor that regulates the expression of various genes connected to DNA repair, apoptosis, stress response, cell cycle arrest, and senescence [58]. The level of p53 is normally kept very low through ubiquitylation by various E3 ligases, mainly Mdm2, that target p53 for proteasomal degradation [59,60]. A plethora of stress conditions induce p53 phosphorylation to block Mdm2 interaction leading to p53 accumulation and expression of its target genes [61]. A variation of this mechanism controls β-catenin activation, the primary player in the Wnt-signaling pathway. In the absence of Wnt signaling, β-catenin is phosphorylated by glycogen synthase kinase-3β (GSK3β), which targets it for ubiquitin-mediated proteasomal degradation. When Wnt engages its receptors, GSK3β is inactivated resulting in β-catenin stabilization, nuclear translocation, and activation of target gene expression [62]. Mechanisms similar to those described for p53 and β-catenin are widespread and employed to control many transcriptional activators. An additional interesting example is the negative feedback circuit mediated by Rpn4 to control proteasome homeostasis in budding yeast [63,64]. Rpn4 is an unstable transcriptional activator that controls transcription of genes encoding proteasome subunits. Rpn4 degradation depends on the proteasome. Consequently, when proteasome activity is limiting, such as during stress conditions or proteasome inhibition, Rpn4 is stabilized, and in turn, promotes the transcription of proteasome subunits to increase the amount of proteasomes [65,66]. Once sufficient cellular proteasome activity is restored, Rpn4 degradation increases and production of proteasome subunits ceases. While the aforementioned Rpn4 dependent mechanism is restricted to yeast, a similar negative feedback mechanism has been shown to exist in higher mammalian cells [67,68] and is at least partially mediated by nuclear factor erythroid-derived 2-related factor 1 [69].

Unlike class A activators, class B and C activators are ubiquitylated when bound to DNA (Figure 2b,c). In addition, degradation of these activators is transcription-dependent. That is, proper assemblyof pre-initiation complex (PIC) and RNA polymerase II recruitment to the promoter is required for the destruction of these activators. Once the transcription complex is assembled, it serves as the substrate recognition component of E3 ligases to promote activator turnover [70]. This so-called *black widow* or *kamikaze model* suggests that activation of transcription marks the activator as “spent” and hence targets it for phosphorylation-dependent ubiquitylation and degradation [70,71]. One well-studied example of this model is the destruction of the yeast activator Gcn4. Gcn4 controls expression of genes involved in amino acid biosynthesis and is polyubiquitylated, followed by proteasome-mediated degradation during transcriptional activation. Gcn4 binds to target promoters, induces pre-initiation complex formation and Pol II recruitment ultimately leading to initiation of transcription. A component of the Pol II holoenzyme, the cyclin-dependent kinase Ssn3/Ssn8 phosphorylates Gcn4 and marks it as “spent”. Initiation of the next round of transcription requires clearing phosphorylated (“spent”) Gcn4 from the promoter, which is achieved by ubiquitylation and degradation by the proteasome [72,73]. This model provides a satisfying explanation for the unexpected observation that activator degradation is required for robust transcription. A similar mechanism has been implicated in activation of other transcriptional activators such as yeast Gal4 [72,73,74] and myc in mammals [75]. The *kamikaze* model also provides a rationale for the observation that transactivation domains frequently overlap with degron sequences [70]. However, it is important to note that this model is not a universal mechanism for transcriptional activation, because there are transactivating factors that are very stable [44,76].

Class C activators require ubiquitylation to complete PIC assembly and their subsequent degradation to allow the polymerase to escape from the initiation phase to elongation while the components of PIC are reassembled for further rounds of transcription (Figure 2c). While direct evidence and a detailed mechanistic understanding for this class of activators remains elusive, this model is consistent with androgen- and estrogen-mediated nuclear receptor signaling. For instance, estradiol binding not only activates transcription of estrogen receptor (ERα) target genes, but also induces proteasome-mediated ERα degradation. Remarkably, proteasome inhibition blocks estrogen-responsive transcription despite ERα accumulation in the nucleus, demonstrating that ERα degradation is necessary for its activity [46,77]. Consistent with the proposed class C model, Reid and colleagues [46] showed that in the presence of estradiol, ERα undergoes cycles of promoter binding and dissociation, and these cycles are dependent on the proteasome.

These different modes of proteasome function in initiation of transcription originally proposed by Lipford and Deshaies [78] provide a useful means of general classification. However, the reader should keep in mind that these mechanisms are not mutually exclusive. Given the complexity of the transcription process and the many signals that converge to control initiation of gene expression, regulation of activators through several of these mechanisms in parallel is likely.

#### 2.1.2. Proteolysis-Dependent Activator Localization

Ubiquitin-dependent degradation of transcription factors does not always lead to complete proteolysis by the 26S proteasome [79]. Transcription factor processing by the 26S proteasome has been well documented for the yeast activators Spt23 and Mga2 [80,81]. Spt23 and Mga2 are homologous activators that control the expression of the fatty acid desaturase Ole1. Spt23 and Mga2 are produced as 120 kDa precursors (p120) and localize to the outer endoplasmic reticulum membrane. Upon fatty acid depletion, homodimerized Spt23 or Mga2 is ubiquitylated by the E3 ligase Rsp5 and one monomer is processed by the proteasome to p90, which remains attached to its precursor binding partner p120 at the ER. In an additional ubiquitin-driven process, the segregase Cdc48 separates p90 from the dimer, initiating nuclear entry of the mature p90 activator resulting in target gene expression [82,83,84]. A variation of this mechanism controls localization of the transcription activator NF-κB in higher eukaryotes [85]. In the canonical NF-κB1 signaling pathway, the 105 kDa precursor is constitutively processed by ubiquitin and proteasome-dependent trimming into the mature 50 kDa NF-κB1 transcription factor (p50) [86,87]. However, p50 is held in a dormant state in the cytoplasm by its inhibitor protein IκBα. Following inflammation, IκBα is phosphorylated, ubiquitylated, and degraded by the proteasome. Destruction of IκBα frees NF-κB to translocate into the nucleus and activate its target genes [81,87]. Proteasome-dependent NF-κB processing plays an even more direct role in noncanonical NF-κB signaling, which is NEMO independent and triggered by some members of the TNF cytokine family and CD40 ligation [88]. The target is the inactive cytoplasmic transcription complex formed by RelB and the p100 precursor of NF-κB2. The C-terminal half of NF-κB2 acts as an inhibitor of the complex and is responsible for cytoplasmic retention. CD40 ligation triggers p100 phosphorylation followed by ubiquitylation and processing into active p52 by the proteasome. Finally, the p52/RelB transcription complex translocates to the nucleus and activates target gene expression [85]. In addition to yeast and humans, proteasome-dependent processing of transcription factors has also been observed for the *Drosophila* protein Cubitus interruptus (Ci), a key regulator of hedgehog signaling [89,90,91]. Mechanistic insight into how proteasomal processing works and how part of the polypeptide is spared from degradation remains scarce. The two proposed models suggest either initiation from the end of the polypeptide [87,92] or an initiating internal proteolytic site that reaches the catalytic core of the proteasome through hairpin loop formation [80]. Tightly folded structural elements in the substrate itself or tight association with binding partners are thought to terminate processing.

Proteasome catalyzed protein processing rather than complete degradation is a well-documented process, but is typically not an activity one associates or anticipates with the 26S proteasome. It may thus be a more common mechanism of transcription factor regulation than the limited known examples suggest.

#### 2.1.3. Turnover of Coactivators and Corepressors by the Proteasome

The regulatory role of the proteasome in transcription is not restricted to its proteolytic activity on activators. Inhibition of proteasomes leads to increased stability of various coactivators including p300, SRC-1 to 3, CBP as well as repressor proteins such as NCoR1 and SMRT. In particular, NCoR1 has recently been identified as a major proteasome target on promoters [93]. Scadden and colleagues [93] used ubiquitin affinity purification and CHIP-seq to map DNA regions that were decorated with increased ubiquitin in response to a pulse of proteasome inhibitors. They reasoned that these genomic regions contain genes especially dependent on proteasome activity. Genomic peaks of proteasome-dependent ubiquitylation correlated well with active genes and particularly with active enhancer and promoter sites. Regions with cyclic AMP response element-binding protein (CREB) were markedly enriched for proteasome-dependent ubiquitylation and a series of experiments deduced that ubiquitylation of the CREB co-repressor NCoR1 contributes to these degradation peaks [93]. NCoR1 mediates the repression of its target genes by associating with histone deacetylases that lead to targeted histone deacetylation [94]. Accordingly, proteasome mediated degradation of NCoR1 has a profound impact on local chromatin structure.

A role of the 26S proteasome in controlling dynamic cofactor exchange is also well illustrated by the example of the LIM homeodomain family of transcription factors (LIM), which promote transcription of genes involved in neuronal development [95,96]. Three additional components control activity of LIM-dependent genes: the coactivator CLIM, the co-repressor RLIM (RING-finger LIM interacting protein), and single-stranded DNA-binding protein 1 (SSDP1). Expression of target genes is activated by the LIM-CLIM-SSDP1 complex, with SSDP1 protecting the complex from recognition by RLIM. Reduced SSDP1 levels allow binding of RLIM, an E3 ubiquitin ligase and co-repressor, which polyubiquitylates the coactivator CLIM, thereby engaging the proteasome to trigger CLIM degradation. RLIM can then insert itself into the LIM transcription complex and repress transcription. In this system, the proteasome initiates an exchange between coactivator and co-repressor [97,98,99]. Many transcription factors control gene expression by assembling multi-protein complexes where the dynamic exchange of components is used for both fine-tuning and as an “on-off” switch. As illustrated by the series of stabilizing and destabilizing interactions in the LIM complex, the proteasome can play an essential role in the plasticity of such systems.

Degradation of DNA bound activators and repressors require their extraction from chromatin. Whether ATPase subunits of the proteasome base can fulfill this activity has not been addressed rigorously. Requirement for the ubiquitin-directed AAA-ATPase Cdc48/p97 prior to engagement of the proteasome has been demonstrated in degradation of an increasing number of DNA-bound proteins [100,101,102,103,104]. Most notably, a critical role of Cdc48/p97 in the release of the ubiquitylated transcriptional repressor α2 from chromatin prior to degradation has been reported [103]. Whether the ubiquitin-directed Cdc48/p97 complex is involved in other degradation events of transcriptional regulators is not known. However, this will be an important question to address, because the involvement of Cdc48/p97 indicates a biochemical step in separating transcription factor ubiquitylation from proteasomal degradation.

### 2.2. Non-Proteolytic Role of the 26S Proteasome in Transcription Activation

**Figure 3:**
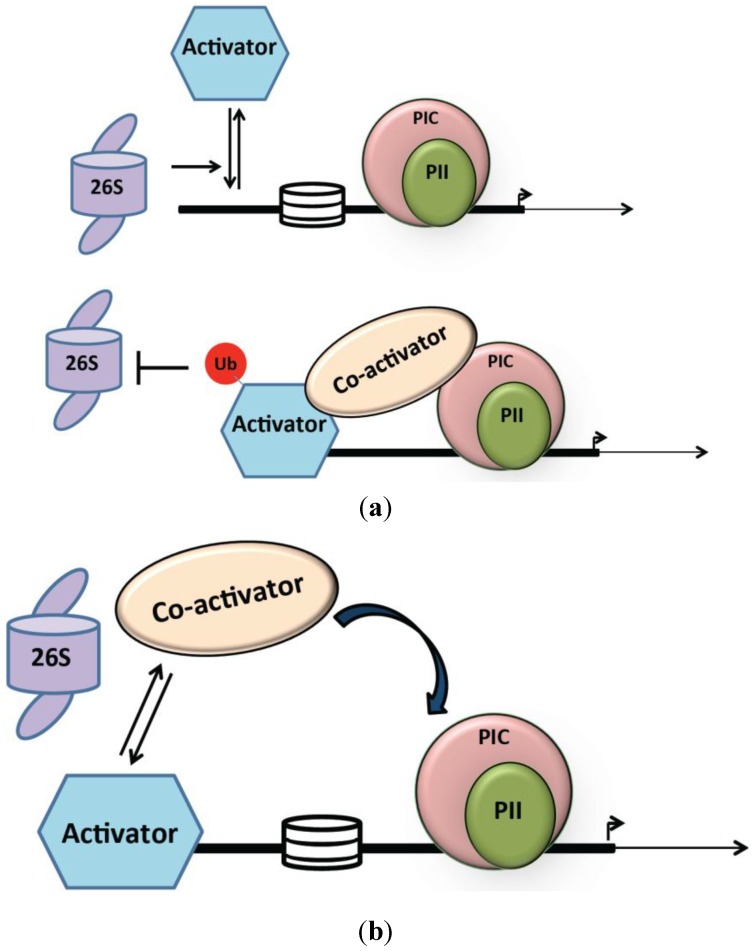
Proteolysis independent roles of the 26S proteasome in activation of transcription. (**a**) 19S components of the 26S proteasome are involved in “stripping” of activators from promoters. Mono-ubiquitylation of the activator renders it resistant to the “stripping activity”; (**b**) 19S subunit of the 26S proteasome stimulates the interaction between activator-coactivator complexes leading to an enhanced association of PIC on the promoter.

Initially viewed merely as destruction machinery, it is now evident that the proteasome is a versatile device with multiple activities. Evidence from studies of proteasome functions in transcription, indicates roles for the proteasome beyond proteolysis. The 19S components, especially the 19S ATPases, promote transcription of genes via non-proteolytic mechanisms that reflect their function as molecular chaperones. As mentioned above, genome-wide location analyses in yeast have mapped 19S and 20S chromatin association sites [50,51]. There is widespread overlap between 19S and 20S subunit binding sites indicating that the 26S particle is located at most active transcription sites. Indeed, recent work from Tansey and colleagues has further substantiated this observation by demonstrating a similar pattern of association and kinetics of both 19S and 20S components during *GAL* gene activation in yeast [105]. However, there are also clearly sites that appear to be occupied exclusively by 19S or 20S subunits [17,50,51,106,107]. Whether these results reflect limitations associated with chromatin immunopurification procedures, such as epitope masking by binding partners, or reveal biologically significant proteasome sub-complexes needs to be addressed. It is however evident from biochemical studies that the 19S complex is sufficient for some transcriptional events such as enhanced coactivator binding [108]. Indeed, the relevant biochemical activity may be contained in the ATPase subunits comprising the proteasome base [17,49,108,109]. Also, biochemical studies have established the interaction of 19S subunits of the proteasome with components of transcription machinery such as RNAPII, activator, and mediator components [108]. Several studies that followed have shed some light on the mechanistic aspects of non-proteolytic roles of the proteasome in transcription activation as discussed below and as depicted in Figure 3.

#### 2.2.1. 19S Mediated Dissociation of Activator-Promoter Complex

Johnston and colleagues’ observation that mutations in 19S subunits (Sug1 and Sug2) could suppress the partial deletion of the Gal4 activation domain (Gal4D) established a link between Gal4 and proteasomal ATPases [41]. It was subsequently shown that the activation domain of Gal4 binds to Sug1 (Rpt6), Sug2 (Rpt4), and other 19S ATPase and non-ATPase subunits. This proteasome complex consisting mainly of 19S ATPases was subsequently termed APIS (AAA+ ATPases of the proteasome independent of 20S) [17,49]. APIS promotes Gal4 dissociation from the promoter in the presence of ATP. Furthermore, such “stripping activity” by APIS was dictated by the ubiquitylation status of Gal4 [49]. Mono-ubiquitylation of Gal4 stabilizes its interaction with the promoter region, while 19S activity destabilizes such an interaction in the absence of mono-ubiquitylation. Accordingly, Gap71, a Gal4 derivative that cannot be mono-ubiquitylated due to a lysine mutation, was efficiently stripped off the promoter by the 19S particle, and a Gal4-ubiquitin fusion was able to escape such stripping activity [49]. These experiments suggest a role of mono-ubiquitylation in protecting the activator against dissociation from the promoter [13]. Similar roles for APIS in activator stripping have been observed at promoters in human cells. For example, mono-ubiquitylation of p53 was suggested to prevent its stripping off the *p21* promoter [110]. However, the exact mechanism of protection from extraction bestowed by mono-ubiquitylation remains elusive and further biochemical studies will be required to understand the mechanism underlying activator stripping.

#### 2.2.2. 19S Proteasome and Activator/Coactivator Interaction

In addition to its role in regulating activator–promoter interactions, the 19S proteasome has been shown to enhance activator–coactivator interaction for efficient PIC formation. In yeast, binding of the activator Gal4 to DNA initiates recruitment of the SAGA (Spt-Ada-Gcn5-acetyltransferase) coactivator, a multi-subunit complex with histone acetyltransferase (HAT) and deubiquitinase activities [111,112,113]. Gal4-SAGA interaction subsequently promotes the assembly of general transcription factors leading to PIC formation. Lee and colleagues reported that the 19S enhances the interaction of Gal4 with SAGA, which depends on 19S ATPase activity, but is independent of the 20S core particle [108]. Additionally, experiments revealed that the 19S base and not the lid is essential for SAGA recruitment. To gain more insight into this process, Lee and colleagues tested whether the 19S proteasome was involved in promoting DNA binding and HAT activity of SAGA. Their studies revealed that the 19S complex physically interacts with SAGA and increases its DNA binding as well as HAT activity. Such an interaction was specific to SAGA as DNA binding or HAT activity of the histone acetyltransferase complex Nua4 was not affected by 19S. These studies clearly establish the non-proteolytic role of 19S in transcriptional activation of SAGA-dependent genes. SAGA regulates transcription of ~10% of yeast genes, and a large fraction of SAGA-independent genes requires TFIID for efficient transcription [9]. Interestingly, a recent study by Uprety and colleagues [114] indicated a similar regulatory role for the 19S particle at TFIID-dependent genes, thereby extending this non-proteolytic function of the proteasome.

Binding of activators to DNA and activator–coactivator interactions are very early steps in transcription. The role of the 19S proteasome in these initial events extends its regulation to sequential steps including PIC formation and transcription elongation. Indeed, biochemical and ChIP analysis from various groups have demonstrated interactions between subunits of 19S and the PIC complex and have also revealed a defective association of PIC components in the absence of 19S proteasome components [106,107,115,116].

An interesting stepwise recruitment of 19S followed by 20S components has also been observed. Similar to yeast genes, the 19S ATPase Sug1 promotes transcription of the MHC class II genes [117]. Human Sug1 and probably other 19S components are recruited to the HLA-DR promoter of MHC class II genes and stabilize the interaction of the relevant class II transactivator (CIITA) with the promoter. Prolonged cytokine stimulation promotes the subsequent recruitment of 20S subunits to the HLA-DR promoter. The inferred slow reconstitution of the 26S proteasome on the HLA-DR promoter coincides with loss of the transactivator CIITA, probably through proteasome-triggered degradation, ultimately repressing MHC class II transcription. A reverse mechanism to this stepwise reconstitution of the 26S complex on promoters with distinct functional consequences has been reported for regulation of HIV-1 transcription [118]. Initially, the 26S proteasome is recruited to the HIV-1 promoter, but transcription becomes productive only after binding of the Tat activator and subsequent recruitment of the proteasome associated protein PAAF1. The latter appears to dissociate the 20S core retaining the 19S particle at the HIV-1 gene where it promotes transcription. These models illustrate the complex and versatile roles of proteasome components in transcription.

The diverse functions and activities associated with the proteasome in regulating transcription prompted Tansey and colleagues [4] to propose the “Swiss army knife” metaphor for the proteasome. In this model, the 26S proteasome is viewed as a protein complex that carries a set of biological functions and biochemical activities that are used by the transcription machinery either individually or in a sequential manner. This useful concept puts less emphasis on the exact composition of recruited sub-particles at individual promoters and is more focused on the biochemical activities performed by the proteasome.

An important but largely unresolved question is how the 26S proteasomes or its sub-components are recruited to promoters. One possibility is attraction by polyubiquitin chains that result from E3 ligase activities on promoters. However, sustained and specific proteasome binding likely requires engagement of additional protein interaction surfaces. Accordingly, both direct proteasome-activator interactions independent of ubiquitin [17,115,119] and specific targeting proteins that tether proteasomes to selected genomic sites have been reported [45,120].

#### 2.2.3. Not All Ubiquitylation of Transcription Components Function through the Proteasome

A final point we would like to make is that the presence of ubiquitin on components of the transcription machinery does not necessarily indicate regulation by the proteasome. The best-studied example is the yeast transactivator Met4. It is regulated by a canonical degradation signal, a K48-linked ubiquitin chain, but in a proteolysis and proteasome independent manner [36,37,76]. Met4 connects metabolic pathways involving sulfur-containing metabolites with cell cycle progression and is maintained in an inactive state by attachment of a K48-linked polyubiquitin chain when nutrients are abundant. The ubiquitin chain represses Met4 transactivation activity through at least two mechanisms. First, the ubiquitin chain recruits the Cdc48/p97 complex, which actively dissociates ubiquitylated Met4 from target promoters [121]. Note that a similar activity has been assigned to the 19S proteasome (see above). Second, the ubiquitin chain has an additional and probably more potent repressive effect on Met4 because transcription of target genes remains largely repressed in *cdc48* mutants despite binding of ubiquitylated Met4 to target promoter regions.

The example of Met4 reminds us that ubiquitylation can have proteasome-independent effects. The involvement of the ubiquitin-directed AAA-ATPase Cdc48/p97 as a reader of the ubiquitin signal suggests that several ubiquitin-triggered pathways can intersect at transcription sites. The coordination and synergy with proteasome-driven events significantly impacts gene expression and requires further mechanistic understanding.

## 3. Conclusions and Future Questions

Our understanding of the roles of the 26S proteasome in transcriptional regulation has increased significantly over recent years. Conceptually it may seem surprising that the machinery responsible for the majority of protein degradation in cells also promotes the synthesis of many of its degradation targets. These multiple functions give the proteasome a major role in sculpting the proteome. In this review, we have discussed various examples of proteolytic and non-proteolytic roles of the 26S proteasome in transcription initiation. While these two roles intersect to mediate a controlled regulation, many questions remain unanswered. What are the mechanisms driving *kamikaze* activators? How does the transcription machinery engage the biochemical activities contained in the 26S proteasome in a temporally and spatially controlled manner? How do transcription sites recruit and attract proteasomes or its sub-particles? What is the role of different ubiquitin chain topologies in transcription? It is likely that answers to these questions will be forthcoming soon.

Studies of proteasome function in transcription have not only expanded our knowledge of the processes governing gene expression, but have also highlighted non-canonical functions of the 26S proteasome. Future research into mechanisms of proteasome function at transcription sites will undoubtedly have important biomedical impacts.
